# Anti-LGI1 encephalitis with initiating symptom of seizures in children

**DOI:** 10.3389/fnins.2023.1151430

**Published:** 2023-04-26

**Authors:** Yang Wang, Dongqing Zhang, Lili Tong, Lu Yang, Ping Yin, Jun Li, Gefei Lei, Xiaofan Yang, Baomin Li

**Affiliations:** Department of Pediatrics, Qilu Hospital of Shandong University, Jinan, Shandong, China

**Keywords:** anti-LGI1 encephalitis, limbic encephalitis, seizures, immunotherapy, children

## Abstract

**Background:**

Anti-leucine-rich glioma-inactivated 1 (LGI1) encephalitis is infrequently reported but more and more recognizable in children. Here we give detailed description of the clinical features and long-term outcome of three cases of childhood onset anti-LGI1 encephalitis.

**Methods:**

Three anti-LGI1 encephalitis patients were hospitalized in the Department of Pediatrics at Qilu Hospital of Shandong University. Data about the clinical manifestations, treatments and long-term follow-up outcomes were described in detail.

**Results:**

Case 1 showed an adolescent girl with initiating symptom of acute-onset frequent focal seizures. Her serum LGI1-antibody test was positive, and she had a good response to antiseizure medication (ASM) and IVIG. Case 2 showed a preschool-age boy with long-period refractory focal seizures and recent behavioral change. Both serum and cerebrospinal fluid (CSF) tests of LGI1-antibody were positive, and the MRI showed progressive atrophy in the left hemisphere. The symptoms got improved after receiving second-line immunotherapy initially but there are still the sequelae of drug-resistant epilepsy and mild to moderate intellectual disability. Case 3 showed an adolescent boy with initiating symptom of acute-onset frequent focal seizures. Both serum and CSF tests of LGI1-antibody were positive, and he had a good response to immunotherapy. By analyzing all literature-reported 19 pediatric cases, we found pediatric anti-LGI1 encephalitis is more common in female and adolescent. Seizures and behavioral changes were the most common symptoms. CSF pleocytosis and LGI1-antibodies results were mostly negative. Most patients showed good response to immunotherapy.

**Conclusion:**

Childhood onset anti-LGI1 encephalitis is a heterogeneous clinical syndrome, ranging from typical limbic encephalitis to isolating focal seizures. It is important to test autoimmune antibodies when encountering similar cases and repeat antibody testing if necessary. Timely recognition leads to earlier diagnosis and more rapid initiation of effective immunotherapy and potentially better outcomes.

## Introduction

Limbic encephalitis is a rare but more and more recognizable immune-mediated disease of central nervous system characterized by neuropsychiatric symptoms including cognitive impairment, memory deficits and seizures (Graus et al., 2016). One of the most common autoimmune limbic encephalitis is anti-leucine-rich glioma-inactivated 1 (LGI1) encephalitis, manifesting as classical limbic encephalitis and also classical faciobrachial dystonic seizures (López-Chiriboga et al., 2018). Lai et al. first discover and confirm that LGI1 is the autoantigen associated with limbic encephalitis previously attributed to voltage-gated potassium channels (Irani et al., 2010; Lai et al., 2010). LGI1 protein is mainly expressed in temporal cortex and hippocampus and mutations in the LGI1 gene cause autosomal dominant lateral epilepsy (Morante-Redolat et al., 2002). Patients with anti-LGI1 antibodies manifest classical limbic encephalitis including seizures, behavior changes, memory deficit, consciousness disturbance and other neuropsychiatric symptoms. Faciobrachial dystonic seizures (FBDS), presenting as stereotyped clonic-like movements of face and ipsilateral limb while lacking electrical correlate, are unique and specific in anti-LGI1 encephalitis but infrequently reported in children. Seizures are commonly reported in both adults and children, though they are rarely reported as the only or initial symptom. Early recognition of immune-related seizures and hence early initiation of immunosuppressive treatment can effectively prevent progression to typical limbic encephalitis and favor better outcomes (Shin et al., 2013). There are only 16 pediatric cases published up to now and each case has its unique characteristics. Here we report three childhood onset cases of anti-LGI1 encephalitis with initiating symptom of seizures, one 11-year-old girl, one 4-year-old boy, and another 17-year-old boy, and the characteristics of clinical manifestation, course of evolution, imaging manifestation and treatment outcomes are summarized, with a review of literature.

## Materials and methods

### Subjects

The study was approved by the Ethics Committee of Shandong University Qilu Hospital. The data of children with anti-LGI1 encephalitis who were hospitalized from July 2019 to September 2022 in Department of Pediatrics of Shandong University Qilu Hospital were collected.

### Methods

Anti-LGI1 encephalitis was first described in 2010 (Irani et al., 2010; Lai et al., 2010) and reached diagnostic consensus in 2016 (Graus et al., 2016). Based on the consensus, diagnosis of anti-LGI1 encephalitis can be made when the following criteria have been met: 1. Acute or subacute onset of working memory deficits, seizures, or psychiatric symptoms suggesting involvement of the limbic system; 2. Presence of positive IgG anti-LGI1 antibodies; 3. Reasonable exclusion of alternative causes. Anti-LGI1 encephalitis was diagnosed by pediatric neurologists at Shandong University, Qilu Hospital on the basis of clinical findings and the presence of specific antibodies in serum or cerebrospinal fluid (CSF).

The serum and CSF samples of each patient were sent to KingMed Center for Clinical Laboratory, Jinan, China, or Dian Medical Laboratory, Hangzhou, China, for the antibodies against the LGI1 and other Autoimmune Encephalitis (AE)-related antibodies. All samples were analyzed by indirect immunofluorescence assay using transfected cells (cell-based assay, CBA) method.

We summarized the symptoms, such as seizures, behavior changes, speech disturbance, sleep disturbance, dyskinesia, movement disorders, consciousness disturbance, memory deficit, and autonomic instability. Clinical data including age, gender, symptoms, CSF analysis, brain magnetic resonance imaging (MRI), electroencephalography (EEG), treatment, and follow-up were collected. Symptomatic treatment included different antiseizure medications (ASMs). First-line immunotherapy included intravenous immunoglobulins (IVIG) or intravenous methylprednisolone (IVMP), or a combination of these. Rituximab or other immunosuppressive drug treatment was defined as second-line immunotherapy. All patients were followed for at least 4 months.

The search strategy of childhood onset anti-LGI1 encephalitis was implemented by the first author and reviewed by another two authors. We searched for literature in PubMed (2000-2023/Feb) using the following terms: (anti-LGI1) OR (anti-leucine-rich glioma-inactivated 1 protein) OR (limbic encephalitis and children) OR (autoimmune encephalitis and children).

## Results

### Case 1

**Figure 1:**
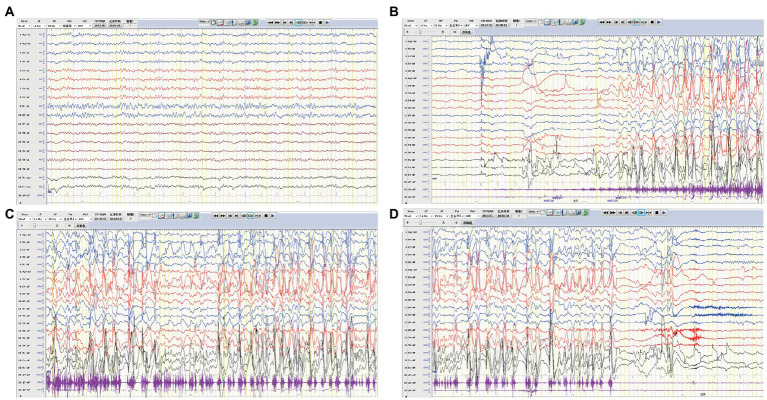
EEG of case 1. **(A)** Slow background activity. **(B–D)** Ictal EEG: a focal seizure originating from the frontal region.

A previously healthy 11-year-old girl presented with acute-onset frequent focal seizures for only 1 day and got admitted to our ward. The seizure initially presented as numbness in the left fingers and then progressed into rhythmic jerking of her left upper limb lasting from 20 s to 1 min each time, during which awareness and memory were retained. She suffered from more than 10 attacks during the whole day. She felt a brief and slight weakness in her left hand with no other complaints. No faciobrachial dystonic seizures were observed. After being admitted to hospital, the seizures repeated like before and were more frequent during sleep. EEG showed slightly slow background activity of 7.5 ~ 8.5 hertz and no obvious interictal epileptiform discharges. The ictal video-EEG captured focal seizures originating from frontal regions with ambiguous laterality, as shown in Figure 1. Brain MRI showed no abnormal signals. Routine blood tests were all negative. CSF tests showed white blood cell count of 2 cells per mm^3^. Serum and CSF were also tested by cell-based assays for a broad panel of neural antibodies. Anti-LGI1-IgG was detected at a titer of 1:300 in serum but negative in CSF. The other tested antibodies against NMDA receptor, AMPAR1, AMPAR2, CASPR2, GABAB receptor, and thyroid antigens were all negative. Tomography scan of thorax and abdomen were negative for tumor. With a presumed diagnosis of anti-LGI1 encephalitis, the patient was treated with oral oxcarbazepine of 0.6 grams per day and intravenous immunoglobulin (IVIG) for 5 days. The seizures got completely relieved after 7 days of treatments. Furthermore, she received a monthly IVIG infusion for 4 times to prevent relapse. After 3 months, the anti-LGI1-IgG in serum turned negative. During a 3-year follow-up, no recurrent seizure and no functional impairment is occurring.

### Case 2

**Figure 2:**
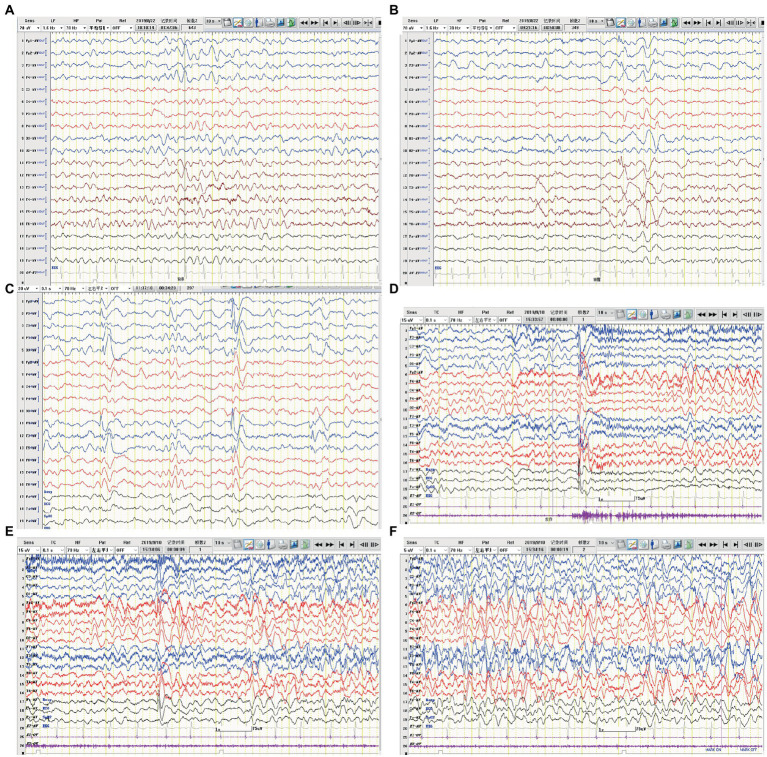
EEG of case 2. **(A)** Slow background activity. **(B,C)** Interictal EEG: epileptiform activity in bilateral frontal and temporal regions. **(D–F)** Ictal EEG: the initial 30 s of a focal seizure originating from the left occipital and posterior temporal regions.

**Figure 3:**
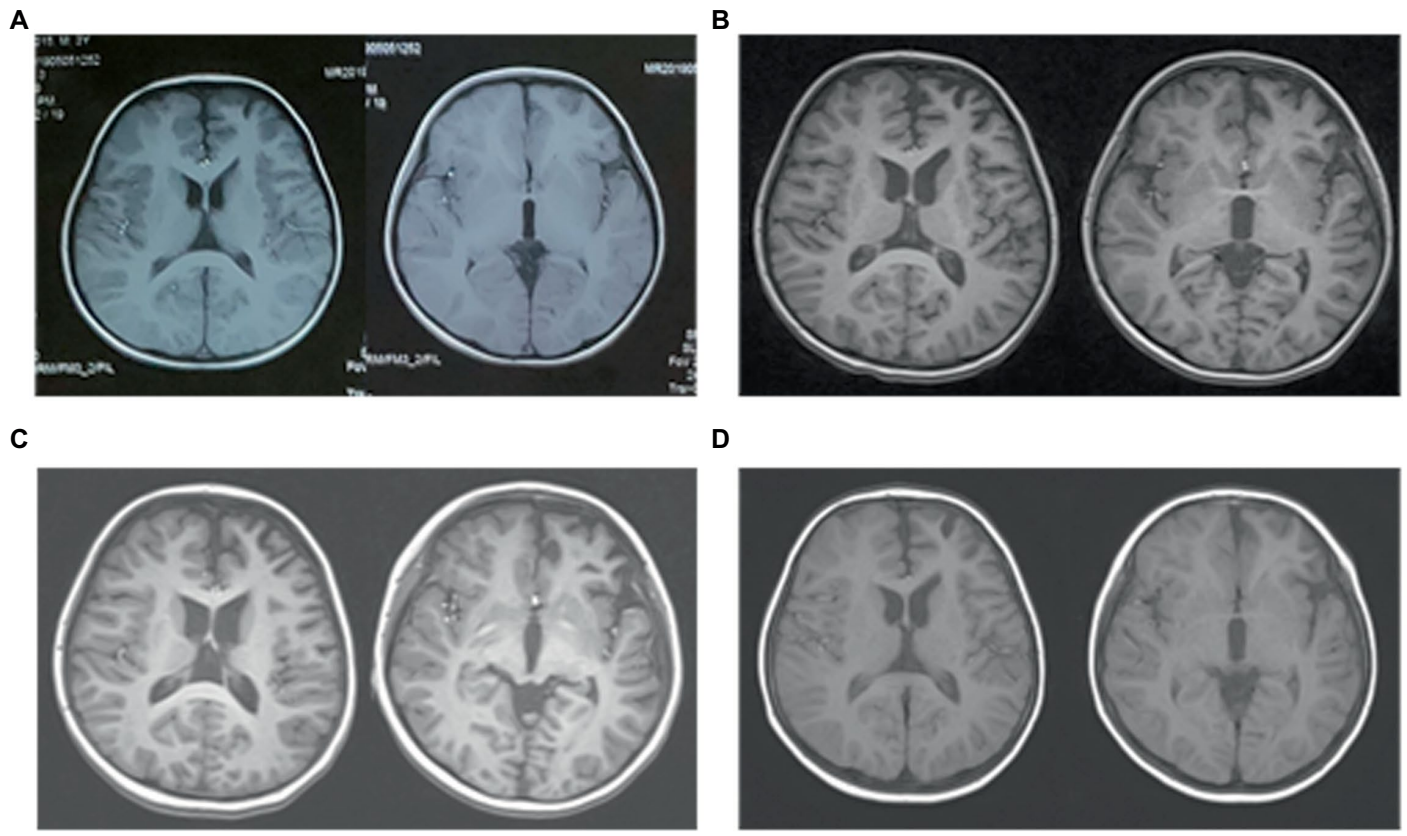
Brain MRI of case 2. **(A)** Three months before admission, axial T1WI shows slightly deeper sulci in left frontal lobe. **(B)** On admission, axial T1WI shows mild atrophy of the left hemisphere, especially frontal and temporal lobe. **(C)** Three months after immunotherapy, there are no obvious progression of cerebral atrophy. **(D)** Follow-up imaging at 9 months after immunotherapy identifies no further damage.

A 4-year-old boy was admitted to our hospital with a history of uncontrolled seizure for nearly 1 year and behavioral change for 2 weeks. The earliest episodes were shown as behavior arrest and blank stare for 5 to 10 s in a frequency of 1–2 times every month. The seizures were infrequent and inconspicuous so they did not cause attention or further treatment at first. 6 months later, changes in semiology and frequency of the seizures were noticed by the parents. They reported one form of episode manifested as behavior arrest during the play or suddenly sit-up during sleep accompanied by a blank stare and drooling with no awareness or responsiveness. Soon after, they found another form of episode manifested as focal tonic–clonic seizures involving the right limbs. Each seizure episode lasted for 30 s to 2 min. He suffered from an increasing frequency of these seizure episodes. He was diagnosed with epilepsy and got started on valproic acid and titrated to a dose of 250 mg twice daily with a weight of 20 Kg. The episodes did not decrease and oxcarbazepine was added. Despite increasing the dose of oxcarbazepine to 450 mg twice daily, he continued to suffer recurrent seizures along with progressive behavioral changes and cognitive impairment for which he was transferred to our hospital for further diagnosis and treatment. On admission, he presented a bad temper of irritability and aggressiveness, showed little interest in toys, and seldom responded to questions. No faciobrachial dystonic seizures were observed. No other abnormal signs were found with a fundamental nervous system examination. Neuropsychological tests revealed global cognitive impairment, especially in memory and executive functions. Before his hospitalization, he had taken the whole exon sequencing test and found no corresponding variation. He also received the tandem mass spectrometry test in blood and gas chromatography mass spectrometry test in urine which ruled out inherited metabolic diseases. EEG showed a slow background activity of 5 ~ 6 Hz and epileptic charges involving bilateral frontal and temporal area, which was more severe in the left hemisphere, as shown in Figure 2. MRI of brain showed mild atrophy on the cortex of left hemisphere, especially frontal and temporal lobes, as shown in Figure 3. No abnormal signal was found in bilateral hippocampus. Routine blood test, viral and bacterial tests, blood ammonia, lactate, homocysteine and urine analysis all showed negative results. CSF tests showed white blood cell count of 4 cells per mm^3^. As for the tests of neural antibodies, anti-LGI1-IgG was detected at a titer of 1:100 in serum and 1:1 in CSF. The other tested antibodies against NMDA receptor, AMPAR1, AMPAR2, CASPR2, GABAB receptor and thyroid antigens were all negative. Tomography scan of thorax and abdomen were negative for tumor. Before we received all related results and made the final diagnosis, we added the third ASM—lacosamide with a titration dose of 100 mg twice daily—to control the seizure but got no significant effect. With a corrected diagnosis of anti-LGI1-encephalitis, the boy was treated with intravenous immunoglobulin with a total dose of 2 g/kg over 4 days and intravenous methylprednisolone with a regimen of 15 mg/kg/d for 3 days with a four-day interval and 3 consecutive rounds in total. The seizure episodes were cut down from more than 10 times daily to 1 ~ 2 times daily, and his behavior, memory and language started improving. Yet not all symptoms were completely controlled. We escalated the treatment into rituximab (375 mg/m^2^ BSA for 4 weeks) plus monthly intravenous immunoglobulin (2 g/kg/round for 5 months) while oral prednisolone was tapered for 6 months. All the symptoms got alleviated gradually but not completely. During immunotherapy, the behavior change disappeared and cognition improved while the seizures gradually decreased to 2 or 3 times per month. Unfortunately, 5 months after receiving rituximab, the seizures gradually became more and more frequent without obvious signs of encephalopathy. A detailed reevaluation revealed no positive findings. The serum anti-LGI1-IgG became negative and brain MRI did not show further atrophy, as shown in Figure 3. The B lymphocyte count remained at 0. The addition of oral azathioprine was rejected by the parents who only accepted the adjustment of ASMs. Gradually adjusting the ASMs regimen, his condition is controlled at a relatively stable level. At the most recent follow-up, 41 months after disease onset, he is still taking valproic acid, carbamazepine, lacosamide, and clonazepam, and the seizures occur once or twice daily. He has mild to moderate intellectual disability and is currently in a special education school.

### Case 3

**Figure 4:**
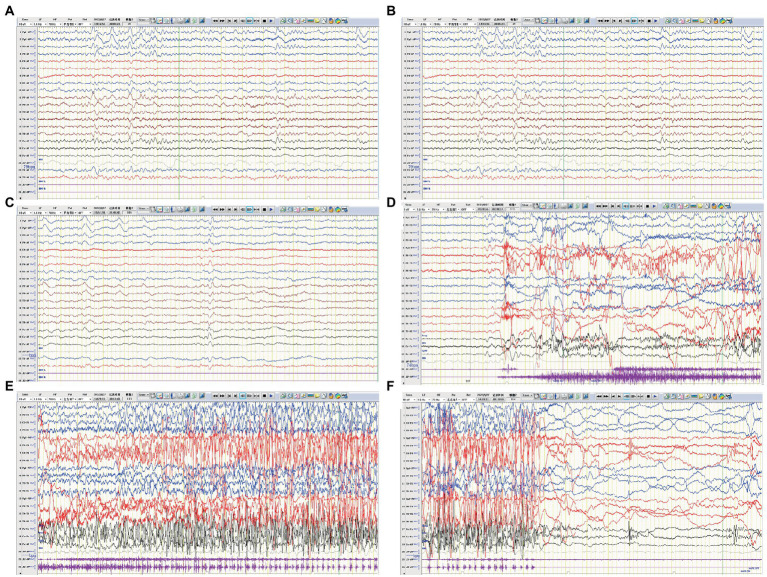
EEG of case 3. **(A)** Slow background activity. **(B,C)** Interictal EEG: slow activity in bilateral frontal regions during both awake and sleep, respectively. **(D–F)** Ictal EEG: a focal seizure originating from the left frontal region.

A previously healthy 17-year-old boy presented with acute-onset frequent focal seizures for 2 days and got admitted to our ward. The seizure presented as loss of consciousness and rhythmic jerking of his right limb, sometimes secondary to bilateral tonic–clonic seizures, each lasting from 30 s to 2 min, occurring approximately 20 times per day. No faciobrachial dystonic seizures were observed. After being admitted to hospital, the seizures repeated like before and were more frequent during sleep. EEG showed slow background activity of 5 ~ 6 hertz, as shown in Figure 4. A few slow waves were observed in bilateral polar frontal, frontal, occipital, and temporal regions during awake. The ictal video-EEG captured focal seizures with impaired awareness originating from the left frontal region. Brain MRI showed no abnormal signals. Routine blood tests were all negative. CSF tests showed white blood cell count of 1 cell per mm^3^. Serum and CSF were also tested by cell-based assays for a broad panel of neural antibodies. Anti-LGI1-IgG was detected at a titer of 1:100 in serum and 1:1 in CSF. The other tested antibodies against NMDA receptor, AMPAR1, AMPAR2, CASPR2, GABAB receptor, MOG-IgG, and thyroid antigens were all negative. Tomography scan of thorax and abdomen were negative for tumor. Valproic acid was administered and tittered to 500 mg twice daily before admission with a blood valley concentration of 83.7 ug/ml but showed no response. Then oxcarbazepine was added and tittered to 0.45 g twice daily. The seizures were reduced to 3–4 times per day. After the presumed diagnosis of anti-LGI1 encephalitis, the patient was treated with intravenous immunoglobulin (2 g/kg over 5 days) and intravenous methylprednisolone (8 mg/kg/d). The seizures were further reduced yet not completely controlled. Intravenous methylprednisolone was initiated with a regimen of 1 g/d for 3 days followed by tapering to oral prednisone. The seizures got completely relieved since the bolus steroid therapy. He has no recurrent seizure or functional impairment during a 4-month follow-up.

## Discussion

Leucine-rich glioma-inactivated 1 (LGI1) was identified as one of the main antigens within the voltage-gated potassium channel complex (VGKC) (Irani et al., 2010). LGI1 protein is mainly expressed in the temporal cortex and hippocampus, where it is secreted into the synaptic space, binding the presynaptic VGKC to the postsynaptic *α*-amino-3-hydroxy-5-methyl-4-isoxazole propionic acid receptor (AMPAR) thus to regulate the signal transduction (van Sonderen et al., 2017). Genetic disruptions of LGI1 that affecting the stability or function of LGI1 protein result in autosomal dominant partial epilepsy, while antibodies against LGI1 can interfere with protein–protein interactions between LGI and ADAMs and cause a diversity of manifestations including limbic encephalitis and seizures (Tominaga et al., 2002; Fukata et al., 2006; Lovero et al., 2015). Further studies documented motor cortex as another major target besides temporal cortex and limbic system in anti-LGI1 encephalitis (Navarro et al., 2016). As with the improvement of recognition and easy access to test technology, more and more cases of anti-LGI1 encephalitis are reported in the recent decade.

The three cases that we are reporting here are totally different in every way including age, gender, characteristics of seizures, neuroimaging tests and responses to treatments. Case 1 was an adolescent girl, case 2 was a preschool-age boy and case 3 was an adolescent boy. After summarizing and analyzing all published data (AlHakeem et al., 2016; Incecik et al., 2016; López-Chiriboga et al., 2018; Schimmel et al., 2018; Zhang et al., 2019; Kang et al., 2022; Wang et al., 2022; Weihua et al., 2022) and these three cases, we found all studies of pediatric anti-LGI1 encephalitis were published in recent years since 2016 and this may due to the increased awareness of the disease in recent years, of which including 9 boys and 10 girls. This female predominance (53%) in a relatively small group is inconsistent with that reported in adults (van Sonderen et al., 2016; Celicanin et al., 2017). The average age was 11 and only 21% (4/19) were in preschool age while 32% (6/19) school age and 47% were in adolescence. Although tests of tumors were negative in all reported cases, regular screening of tumors should remain a priority, because the onset age was predominantly in older children and the lack of long-term follow-up results. Tables 1, 2 show the cumulative clinical data and characteristics of 19 cases with pediatric anti-LGI1 encephalitis.

**Table 1:** Cumulative clinical data of 19 cases of children with anti-LGI1 encephalitis.

Case	Gender/Age (years)	Symptom	Brain MRI	EEG	Serum LGI1-Ab	CSF tests	Tumor screening	Treatment	Outcome	Ref
1	F/11	Focal seizures	Normal	slow background activity without epileptiform discharges	1:300	Normal	Negative	OXC, IVIG	Seizure cessation, back to school at 3-year follow-up	From this article
2	M/4	Refractory focal seizures, behavior change	Atrophy of cortex in left hemisphere	slow background activity with epileptiform discharges	1:100	Positive for LGI1-Ab (1:1)	Negative	Symptomatic, IVIG, IVMP, Pred, Rituximab	Improved seizure control, improved encephalopathy, go to special education school	From this article
3	M/17	Focal seizures	Normal	slow background activity with slow discharges	1:100	Positive for LGI1-Ab (1:1)	Negative	VPA, OXC, IVIG, IVMP, Pred	Seizure cessation, back to school at 4-month follow-up	From this article
4	M/9	Encephalopathy, frequent focal seizures, paroxysmal dizzy spells, hypersomnia, behavioral changes	Brainstem: T2 hyperintensity without enhancement	Right temporal spikes and sharp waves	Positive	Normal	Negative	IVIG	Improved seizure control, improved encephalopathy, back to school	López-Chiriboga et al. (2018)
5	F/17	Rapidly progressive severe disabling bilateral lower extremity pain, cramps and dysautonomia, encephalopathy, prominent insomnia	Normal	Not provide	Positive	Normal	Negative	IVIG	Improved neuropathic pain	López-Chiriboga et al. (2018)
6	F/17	Influenza-like symptoms followed by encephalopathy and frequent focal seizures with left arm paresthesias	Normal	Several focal right and left temporal and left frontal epileptiform discharges	Positive	OBs positive	Negative	IVIG, AZA	Improved, back to school	López-Chiriboga et al. (2018)
7	F/5	Hypersomnolence, decline in school performance, frequent focal seizures with left arm paresthesias followed by bilateral upper extremity posturing, multiple episodes daily	Normal	Epileptiform bifrontal discharges	Positive	Normal	Negative	VPA, LCS	Seizure cessation with ASMs, back to school	López-Chiriboga et al. (2018)
8	M/13	Encephalopathy, amnesia, neuropsychiatric symptoms, new temporal lobe seizures, neuropathic pain affecting lower extremities	Left limbic T2 hyperintensities	Normal	Positive	Pleocytosis (22/mm^3^)	Negative	IVIG, IVMP	Improved, back to school	López-Chiriboga et al. (2018)
9	F/17	Profound weight loss, dysautonomia, intractable abdominal pain, constipation, gastroparesis, cramps	Not provide	Not provide	Positive	Not provide	Negative	Symptomatic	Not available	López-Chiriboga et al. (2018)
10	F/14	History of ASD, paroxysmal dizzy spells, neuropsychiatric symptoms, cramps	Normal	Normal	Positive	Not provide	Negative	Symptomatic	Stable	López-Chiriboga et al. (2018)
11	M/8	Sleep disorder	T2 hyperintensities in left hippocampus	Normal	Positive	Positive for LGI1-Ab	Negative	IVIG, Pred	Improved	Zhang et al. (2019)
12	M/15	Seizures	Normal	Not provide	1:100	Normal	Negative	IVIG, LEV	Stable and no relapse at one-year follow-up	Zhang et al. (2019)
13	M/14	Memory deficit, behavior change, episodes with half-side pallor and paraesthesia of the face with ptosis	T2 hyperintensities in bilateral hippocampus	Normal	1:1000	OBs positive, negative for LGI1-Ab	Negative	IVMP, PE, Pred, MMF	Improved. Memory and behavior impairment did not completely reversible at 5-year follow-up	Schimmel et al. (2018)
14	F/7	Psycho-behavioral changes, sleep disorders, gatism, perioral movements	Normal	slow background activity without epileptiform discharges	Positive	Not provide	Negative	IVIG, IVMP	Stable	AlHakeem et al. (2016)
15	F/8	Behavioral changes, hallucinations, confusion, and complex partial with secondary generalized seizures	T2 hyperintensities in right medial temporal lobe	Epileptiform activity in right frontotemporal region	Positive	Normal	Negative	LEV, IVIG, IVMP, Pred	Improved	Incecik et al. (2016)
16	F/7	Focal seizures	Normal	slow background activity with epileptiform activity	1:30	Normal	Negative	OXC, IVIG, IVMP, Pred	Seizure-free at 2 years follow-up	Wang et al. (2022)
17	M/2.7	Cerebellar ataxia including progressive unsteady gait and tremor	Normal	Not provide	1:32	Normal	Negative	IVMP, Pred	Stable and no relapse at 4-year follow-up	Weihua et al. (2022)
18	M/4	Dysarthria, uncontrollable seizures, progressive ataxia, irritability and insomnia, paroxysmal movement disorder, FBDS	Not provide	Diffuse slow waves	1:100	Positive for LGI1-Ab (1:10)	Negative	LCM, IVIG, IVMP, Pred	Stable and no relapse at 10-month follow-up	Weihua et al. (2022)
19	F/13	Status epilepticus, behavioral symptoms, disturbance of consciousness, memory deficit, speech disorders, sleep disorder, headache, fever, FBDS	Abnormal findings in temporal lobe	Slow waves and epileptic discharges	Positive	CSF pleocytosis (>5/mm^3^), positive for LGI1-Ab	Negative	IVIG, IVMP	Recovered after treatment	Kang et al. (2022)

Case 1-Case 3 come from this article and Case 4-Case 19 come from published literature. M, male, F, female, EEG, electroencephalogram, CSF, cerebrospinal fluid, IVIG, intravenous immunoglobulin, IVMP, intravenous methylprednisolone, OBs, oligoclonal bands, AZA, acetazolamide, MMF, mycophenolate mofetil, LCM, lacosamide, VPA, valproate acid, LEV, levetiracetam, OXC, oxcarbazepine, CBZ, carbamazepine, CZP, clonazepam, Pred, prednisone, PE, plasma exchange.

**Table 2:** Patient characteristics of 19 cases of children with anti-LGI1 encephalitis.

Characteristics	Number (%)
Female	10/19 (53%)
Age at onset (years)	11 (range 2.7–17)
Preschool age	4/19 (21%)
School age	6/19 (32%)
Adolescence	9/19 (47%)
Symptom during whole disease course	
Seizures	12/19 (63%)
Behavior changes	8/19 (42%)
Sleep disorder	6/19 (32%)
Encephalopathy	6/19 (32%)
Dysautonomia	3/19 (16%)
Memory deficit	3/19 (16%)
Movement disorder	2/19 (11%)
Cerebellar ataxia	2/19 (11%)
Speech disorder	2/19 (11%)
FBDS	2/19 (11%)
MRI results provided	
Normal	10/17 (59%)
Abnormal	7/17 (41%)
Temporal lesion	5/17 (29%)
Frontal lesion	1/17 (6%)
Brainstem	1/17 (6%)
EEG results provided	
Normal	4/15 (27%)
Abnormal	11/15 (73%)
Slow background activity	7/15 (47%)
Interictal epileptiform discharges	8/15 (53%)
CSF results provided	
Normal	8/16 (50%)
Abnormal	8/16 (50%)
Pleocytosis	2/16 (13%)
for LGI1 antibodies	5/16 (31%)
Positive for OBs	2/16 (31%)
Tumor screening	
Positive	0/19
Treatment	
Symptomatic only	3/19 (16%)
IVIG/PE	15/19 (79%)
IVMP	10/19 (53%)
AZA/MMF	2/19 (11%)
Rituximab	1/19 (5%)

Seizures, especially focal seizures, are common symptoms of anti-LGI1 encephalitis in both pediatric and adult age. All our cases presented with focal seizures but in different forms and frequencies. According to the ILAE 2017 operational classification of seizure types (Bing-Lei et al., 2019), case 1 initially presented with paresthesia in the left fingers and then progressed into rhythmic jerking of the left upper limb, which was classified as focal sensory seizure progressing to clonic left arm jerks. Case 2 exhibited two kinds of seizure types, focal non-motor onset seizure with behavior arrest and impaired awareness at first, and focal motor onset clonic seizure with impaired awareness in progressive stage. Case 3 manifested as loss of consciousness and rhythmic jerking of his right limb while sometimes secondary to bilateral tonic–clonic seizures, which was classified as focal clonic seizures with impaired awareness with or without progressing to bilateral tonic–clonic seizures. We did not observe FBDS in these three cases and so far, there are only two documented cases of FBDS in children (Kang et al., 2022; Weihua et al., 2022). More than half of the pediatric cases (63%, 12/19) presented with seizures, of which 9 cases that provided detailed data showed focal seizures. Electroencephalogram results were heterogeneous. Although seizure episodes were frequent, 7 cases showed no interictal epileptiform discharge and the other 8 cases exhibited frequent discharge. Based on adult data, response percentages of most prescribed ASMs in patients with anti-LGI1 encephalitis were not satisfactory while carbamazepine appeared more effective than valproate acid or levetiracetam (de Bruijn et al., 2019). The seizures were controlled with ASMs alone in one patient and the other 11 patients received immunotherapy or immunotherapy combined with ASMs to get seizures controlled partially or completely. Yet, more case studies are needed to identify the characteristics of seizures and EEG in order to aid early diagnosis and interventions.

Behavior change is another notable symptom that presented in 42% of reported pediatric cases, which was also exhibited in case 2. It is recognized as a caution of limbic encephalitis (Graus et al., 2016) and showed good response to immunotherapy in most reported cases (AlHakeem et al., 2016; Incecik et al., 2016; López-Chiriboga et al., 2018). Beyond that, memory deficits and sleep disorders were also frequently reported in pediatric anti-LGI1 encephalitis, which were consistent with adult cases (van Sonderen et al., 2016; Fisher et al., 2017; López-Chiriboga et al., 2018; Kang et al., 2022).

As for CSF tests, no pleocytosis or oligoclonal bands were detected in our cases, but in case 2 and case 3, the anti-LGI1 antibody were positive in a low level of titer. Combined with other pediatric cases, either pleocytosis or oligoclonal bands were frequently detected in CSF. Of the total 16 cases providing CSF data, 50% (8/16) of the children were negative, while only 2 cases showed CSF pleocytosis, 2 cases were positive for OBs, and 5 cases showed positive results for anti-LGI1 antibodies. The results were in accordance with previous studies in adult anti-LGI1 encephalitis (Jarius et al., 2008; Graus et al., 2016). Thus, we should keep these tips in mind that the absence of pleocytosis does not rule out limbic encephalitis and normal routine CSF tests do not imply the absence of CSF antibodies. These tips can also apply to analyze the neuroimaging results. Case 2 showed atrophy of cortex in frontotemporal lobe while case 1 and case 3 showed normal MRI. Compared with reports of literature, 59% of pediatric patients showed normal brain MRI and for those with abnormal results, the majority showed lesions of temporal lobe and hippocampus, which were also in accordance with adult anti-LGI1 encephalitis. Progressive cerebral atrophy was reported only in one adult case (Yelam et al., 2019). However, Szots et al. (2017) found the existence of progressive global brain atrophy in patients with LGI1 limbic encephalitis, especially in temporal limbic structures, frontal lobe and the cerebellum, using a volumetric analysis method of the T1-weighted MRI data, despite early immunotherapy. This was attributed to neuroinflammation as well as metabolic changes, like abnormal glutamine and glutamate levels detected with MR spectroscopy. This further underscores the importance of early and vigorous immunotherapy.

Two of our cases with short medical history before admission responded well to symptomatic therapy combined with immunotherapy at long-term follow-up, and the other one with a quite long history responded initially to first-line plus second-line immunotherapy but eventually suffered sequelae of drug-resistant epilepsy and intellectual disability. Nevertheless, we believe that it is not the anti-LGI1 encephalitis that is not well controlled, but the static structural brain damage left after treatment, the atrophy in the left frontotemporal lobe, that result in structural drug-resistant epilepsy and intellectual disability. Consistent with the literature in children (Zhang et al., 2019; Wang et al., 2022), when acute onset seizures are the solitary symptom of anti-LGI1 encephalitis, the seizures are well controlled after timely treatment. Although only a few similar cases have been reported, we can expect that if the boy in case 2 had received a timely diagnosis and effective immunotherapy in the onset of the encephalitis, he might have had the same outcome as the other two cases. Almost all reported cases of pediatric anti-LGI1 encephalitis showed relatively good outcomes and went back to school smoothly, although part of them were receiving long-term immunosuppressants or steroids. Compared with anti-NMDAR encephalitis, which is a more common autoimmune encephalitis in children, patients seem to respond faster and better to immunotherapy (de Bruijn et al., 2018). As for adults, patients with anti-LGI1 encephalitis do show better long-term outcomes and lower recurrent rates than those with anti-NMDAR encephalitis (Malter et al., 2014; Finke et al., 2017). Yet we still need long-term follow-up results to evaluate the prognosis in children.

We still have doubts about the course of ASMs in all cases. In most patients, seizures resolve after autoimmune encephalitis subsides in the nationwide cohort study and ASMs are recommended to be a functional add-on treatment (de Bruijn et al., 2019). However, the overall effect of ASMs in the symptomatic treatment of seizures is limited and antibody-dependent. Therefore, ASMs are not recommended as primary and long-term treatment (Shin et al., 2018; de Bruijn et al., 2019). As for case 1, we recommended reducing the dose gradually at 1-year follow-up. But the patient and her mother were so worried about the possibility of seizure relapse, they even wish to continue the medication even with seizure free for 2 years. For those cases presenting with ASMs-effective seizures, the course of treatment is an issue worthy of further studies.

There are some limitations in our study. Most importantly, the sample size is small. Anti-LGI1 encephalitis is rare in children which makes it difficult to perform a stratified analysis. Besides, in case 3, the change in antibody level is not available. The patient did not undergo planned antibody testing because of bad compliance.

Overall, we report three childhood onset cases of anti-LGI1 encephalitis with initiating symptom of seizures, and the characteristics of clinical manifestation, course of evolution, imaging manifestation and treatment outcomes are summarized, with a review of literature. Comparing with adult-onset anti-LGI1 encephalitis, which existing a pathognomonic manifestation like FBDS, pediatric anti-LGI1 encephalitis is infrequently reported and shows little similarities in clinical manifestations except typical symptoms associated with limbic encephalitis (Sen et al., 2014; van Sonderen et al., 2016). We assume that early recognition of immune-related seizures and hence early initiation of immunosuppressive treatment may prevent progression to some extent, and favor better outcomes. We have to acknowledge the difficulties in early identifying and diagnosing limbic encephalitis including anti-LGI1 encephalitis in children, due to the complexity and diversity of clinical manifestations and the atypia and dynamic change of related testing results. With accumulative clinical experience and better access to antibody detection methods, we hope to reach a diagnostic consensus on limbic encephalitis and anti-LGI1 encephalitis in children to help guide clinicians to make sensible evaluations, diagnosis and tailored treatments.

## Data availability statement

The raw data supporting the conclusions of this article will be made available by the authors, without undue reservation.

## Ethics statement

Written informed consent was obtained from the minor(s)’ legal guardian/next of kin for the publication of any potentially identifiable images or data included in this article.

## Author contributions

YW and XY designed the study. YW wrote the first draft. DZ, LT, LY, PY, and JL collected the data and fulfilled data analysis. GL and BL contributed to the conception of the work and revised it critically for important intellectual content. XY revised this manuscript and approved for the submission. All authors contributed to the article and approved the submitted version.

## Funding

This project was supported by Natural Science Foundation of Shandong Province (grant no. ZR2021MH229). Gratitude to the patients and parents for their understanding and cooperation.

## Conflict of interest

The authors declare that the research was conducted in the absence of any commercial or financial relationships that could be construed as a potential conflict of interest.

## Publisher’s note

All claims expressed in this article are solely those of the authors and do not necessarily represent those of their affiliated organizations, or those of the publisher, the editors and the reviewers. Any product that may be evaluated in this article, or claim that may be made by its manufacturer, is not guaranteed or endorsed by the publisher.
